# Takayasu’s Arteritis with Subcutaneous Nodules in a 4-year-old Child: A Case Report

**DOI:** 10.31729/jnma.5387

**Published:** 2020-11-30

**Authors:** Sunil Kumar Das, Aakrit Dahal, Nikhil Shrestha, Sajal Tnawanasu, Subash Sharma

**Affiliations:** 1 Patan Academy of Health Sciences, Lalitpur, Nepal; 2 Oxford University Clinical Research Unit-Nepal, Lalitpur, Nepal

**Keywords:** *child*, *subcutaneous nodules*, *Takayasu's arteritis*

## Abstract

A 4-year-old girl who presented with pain in the abdomen, subcutaneous nodule, fever and was later diagnosed with Takayasu arteritis. Oral corticosteroid and methotrexate were started. Childhood TA should be kept in differential diagnosis when presented with subcutaneous nodules and increased acute phase reactants.

## INTRODUCTION

Takayasu's arteritis, also known as pulseless disease, is a rare form of an inflammatory disease involving the inner walls of the larger arteries in the body, the aorta and its major branches. Takayasu's arteritis (TA) is a chronic vasculitic disease of unknown etiology. Clinically significant renal disease is relatively common, and renovascular hypertension is the major renal problem.^1^ It results from an inflammation of the artery wall with predominance of CD4+ T lymphocytes and macrophages which results in inflammation induced vascular remodeling leading to intimal hyperplasia and stenosis. Early Diagnosis of TA remains challenging due to non-specific symptoms.

## CASE REPORT

A 4-year-old girl presented with pain abdomen for 1 day and subcutaneous nodules for 3 days. Pain abdomen was acute in onset in the left hypochondrium, intermittently present, non-radiating, and associated with abdominal fullness with no aggravating or relieving factors. Additionally, subcutaneous nodules were also present on anterior aspect of forearm, medial aspect of both thighs and over abdomen with initial bluish discoloration later followed by visible nodules which were painful and fixed and there was no discharge. Subcutaneous nodules appeared intermittently for last 3 months and recently appeared for 3 days, she had no history of headache, altered consciousness, abnormal body movements, visual disturbances, photophobia, decreased vision, nausea, vomiting, altered bowel habit, dysuria, hematuria, decreased urine output, fast breathing or labored breathing, skin rashes, joint swelling or bleeding manifestation from any sites. No history of medication taken for this illness, no recent travel history or exposure to COVID positive case or suspected individuals.

After admission; on examination the child was irritable, looked pale, there was facial puffiness, lymphadenopathy, with fever of 100.4^0^F, respiratory rate of 42 breaths per minute, and pulse of 152 beats per minute, which was palpable, good in volume with decreased pulse volume in upper limb on left side. Blood pressure was 160/78 mm of Hg on left arm and 140/60 mm of Hg on right arm which was >99^th^ centile for height for age i.e. stage 2 hypertension.

**Figure 1:**
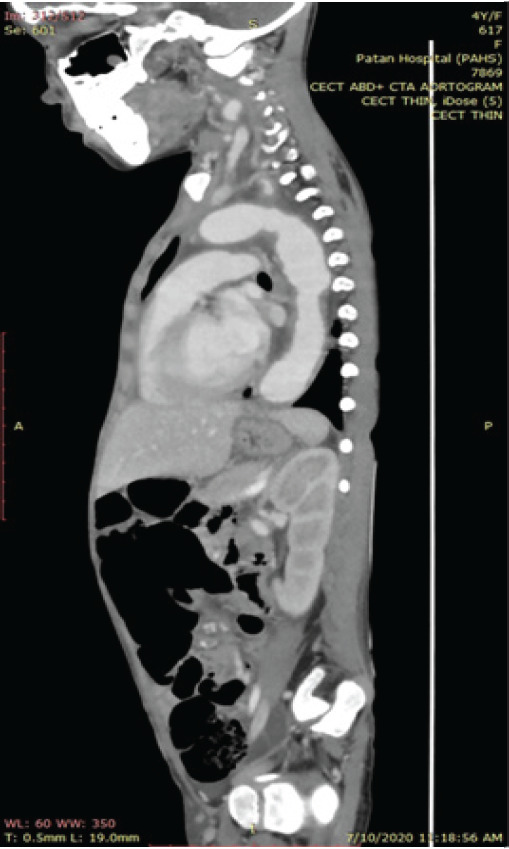
CECT thorax and abdomen sagittal view.

**Figure 2:**
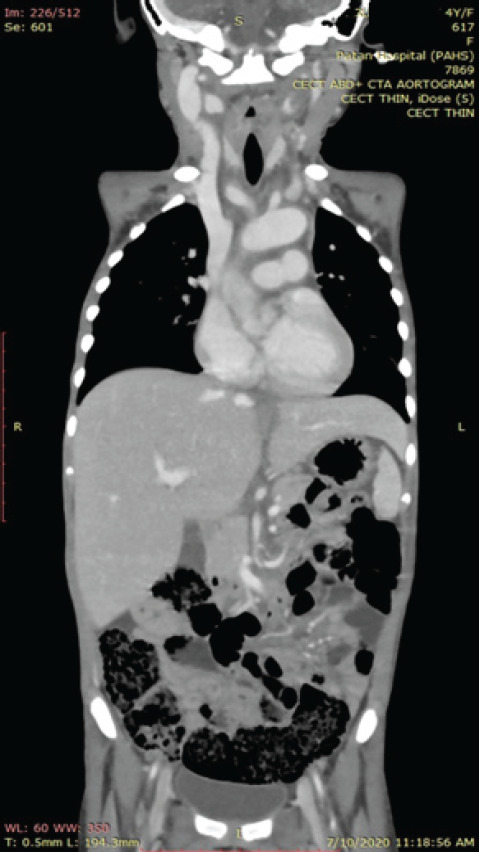
CECT thorax and abdomen coronal view.

Keeping in the mind the differential diagnoses of malignancy, vasculitis, juvenile rheumatoid arthritis, tuberculosis, systemic lupus erythematosus, typhoid, infectious mononucleosis, brucellosis and leptospirosis needful laboratory investigation and imaging were sent. Her bone marrow showed normal picture. Rheumatoid factor and ANA were negative. Her blood cultures were negative despite of persistent fever without focus. Liver function and renal function test were within normal limits. Her mantoux test was negative. She had widened mediastinum seen in chest x ray. As per protocol of fever of unknown origin, to find out the cause of the fever; CT thorax and abdomen was advised, and the findings were suggestive of takayasu arteritis (Figure 1,2). It was accompanied with the positive findings of diffuse transmural wall thickening with luminal irregularity involving the aorta and its branches, aortic arch, descending thoracic and abdominal aorta up to its infra renal segments, brachiocephalic trunk, bilateral common carotid, subclavian and axillary arteries, celiac trunk, superior mesenteric artery, bilateral renal arteries, common femoral arteries and pulmonary arteries. All features were suggestive of vasculitis likely takayasu arteritis (type V). Mild stenosis involving mid segment of right renal artery and hypodensities at right renal cortex suggestive of renal infarction were also observed. Fusiform aneurysm involving aortic arch and descending thoracic aorta as well as luminal irregularities involving descending thoracic and abdominal aorta were seen. Small collection at both upper anterior wall likely haematomas as well as minimal ascites was found on imaging.

Her laboratory reports are shown (Table 1). Other reports including Peripheral blood smear showed anisopoikilocytosis, microcytic hypochromic erythrocytes, few pencil cells; leukocytosis with normal morphology; thrombocytosis; but there was no atypical cells and parasites on the smear. Her Echocardiography showed left ventricle ejection fraction (LVEF) of 60%; mild tricuspid regurgitation with mild pulmonary hypertension (32+5=37 mm of Hg) without regional wall motion abnormality (RWMA). There was no intracardiac mass or thrombosis.

**Table 1:** Laboratory parameters

Lab Parameters	On the day of admission
Total Leukocyte count (/μl)	33,600
Differentials (%)	N 85 L15
Platelet (/μl)	645*103
Hemoglobin (g/dl)	7.9
Urea (mg/dL)	14
Creatinine (mg/dL)	0.2
Sodium (mmol/L)	136
Potassium (mmol/L)	4
Calcium (mg/dL)	7.9
Magnesium (mg/dL)	2.3
^*^CRP (mg/l)	362
^*^ESR (mm/hr)	84
^*^PT/ INR	19/1.48
^*^APTT (secs)	64

* PT: Prothrombin time; INR: International normalizedratio; APTT: Activated partial thromboplastin time;ESR: Erythrocyte sedimentation rate; CRP: C-reactiveprotein.

She was initially managed in the line of infection and lymphoma. Later she developed fever and hemodynamic instability on the second day of admission for which she was admitted in ICU for close monitoring of blood pressure. She stayed in ICU for 24 hours and was again shifted to ward. Her cause of fever was not found. Later CECT and CT angiogram were done on day 7 of admission, which was suggestive of TA. She was then treated with antihypertensive medication, oral steroid and methotrexatefor her condition and to prevent further complications. Before discharge she stayed in hospital for 11 days and was asked to follow up after 4 weeks. She was planned to tapper down corticosteroid and optimize antihypertensive drugs on follow up.

## DISCUSSION

Takayasu's disease is a chronic inflammatory disease of large and medium-sized arteries, involving the aorta and its main branches, the pulmonary arteries and the coronary tree. Since the original report of Takayasu's disease in 1908,^2^ but recently all medications were discontinued because of an episode of syncope and a finding of very low arterial pressure (95/60 mmHg in the right arm. The incidences of TA were estimated to be 1-2 per million in Japan and 2.2 per million in Kuwait. Recent epidemiologic studies suggest that TA is being increasingly recognized in Europe with reported incidence estimate varying from 0.4 to 1.5 per million.^3^ Childhood TA is a devastating disease with mortality rate as high as 35%.^4^ The clinical manifestation of TA varies according to duration along with the course of disease. In acute inflammatory phase, it is often unrecognized and may be characterized by constitutional symptoms like anorexia, fatigue, night sweats, weight loss, arthralgia and skin rashes lasting from weeks to month with relapsing and remitting course. In the chronic phase of the disease, stenosis develops and features secondary to arterial occlusion become clinically overt.. The majority of patients presents with increased inflammatory markers including CRP and ESR. While the sensitivity of these markers is high for active TA, they lack specificity.^5^

The European League Against Rheumatism (EULAR) endorsed criteria for pediatric vasculitides was validated at the 2008 Ankara Consensus Conference and the final version of the classification criteria for each of the vasculitides was published in 2010 as the EULAR/Pediatric Rheumatology International Trials Organization (PRINTO)/PRES criteria as depicted (Table 2). This is currently being used to classify c-TA with a sensitivity and specificity of 100% and 99.9%, respectively.^6^

**Table 2:** PRES criteria.

Criteria	Definition
Angiographic abnormality	Angiography (conventional/CT/MRI) of aorta or its main branches and pulmonary arteries - aneurysm or dilatation, narrowing, occlusion or thickened arterial wall not due to fibromuscular dysplasia or similar causes
Plus, one of the following five	
Pulse deficit or claudication	Lost/decreased/unequal peripheral artery pulse(s) Or Claudication: focal muscle pain induced by physical activity
Blood pressure difference >10 mmHg	Difference of >10 mmHg in systolic blood pressure between arms
Bruit	Audible murmurs or palpable thrills over large arteries
Hypertension	Systolic/diastolic BP greater than 95th centile for height
Acute phase reactants	Erythrocyte sedimentation rate > 20 mm per first hour or CRP any value above normal

Most important aim of treatment is controlling inflammatory process, preventing secondary end organ damage and controlling hypertension. The most commonly used agents include corticosteroids and conventional immunosuppressive agents such as MTX, AZA, MMF and LEF.^7^ The most commonly used agents include corticosteroids and conventional immunosuppressive agents such as MTX, AZA, MMF and LEF. In patients who remain resistant and/or intolerant to these agents, biologic drugs including TNF inhibitors, rituximab and tocilizumab seem to be promising. Antiplatelet treatment may also lower the frequency of ischaemic events in TA. In the presence of short-segment, critical arterial stenosis, balloon angioplasty or stent graft replacement may be useful. On the other hand, long-segment stenosis with extensive periarterial fibrosis or occlusion requires surgical bypass of the affected segment, which is clearly associated with superior results compared with endovascular intervention. As a general rule, both endovascular intervention and surgical procedures should be avoided during the active phase of the disease. Earlier diagnosis, better assessment of disease activity and future clinical trials will obviously improve the management of TA. Hence, the patient was started on corticosteroid and methrotrexateand other secondary medication for hypertension. Approximately 60% to 80% of patients treated with glucocorticoids have been found to achieve symptomatic relief.^8^ Our patient responded with first dose of steroid with remission of fever, and decrease in ESR on subsequent days was noted as well.

In a study carried out by Aeschlimann et. al. adverse outcomes were documented in 14 (52%) children: two (7%) died within 6 months of diagnosis, and 13 (48%) experienced disease flares. The 2-year flare-free survival was 80% with biologic treatments compared to 43% in non-biologic therapies (p=0.03); at last follow-up, biologic therapies resulted in significantly higher rates of inactive disease (p=0.02).^4^ In South Africa, the mortality rate was 22.5%; 7 of 31 patients died because of hypertension or complications after kidney transplantation.^9^ Prevention of organ damage may avoid worse outcome.

A study of childhood TA by Fan et.al. reveals an early mortality and morbidity, with 3% of patients dying by the first year and around 50% enduring at least an event or re-hospitalization within the first 5 years after diagnosis.^10^ There are few follow-up studies in children, and the mortality rate has ranged from 21% to 40% in the short term.^9^ More recently, the prognosis has significantly improved due to interventional procedures for the treatment of renal and aortic stenosis.

Takayasu's arteritis is a rare form ofvasculitis in pediatric population which has to be considered in differential diagnosis for any case with unexplained hypertension, subcutaneous nodules and pulse difference. Thorough history, examination and investigation can lead to correct diagnosis. Since the disease can be severe, rapidly progressive and life threatening, an early recognition is crucial in order to start medication and to prevent morbidity.
